# How Poisons Were Tried on Man

**Published:** 1909-07-31

**Authors:** 


### HOW POISONS WERE TRIED ON MAN.

From a very early period science has been gradually built up by experimental methods, and even the ancients were cognisant of the fact that the remedial properties of substances could only be proved by actual experiment. Not only animals but human beings were utilised for this purpose by many famous physicians in the Middle Ages. Criminals who had been condemned to death were generally selected for these experiments.

Vivisection of the live human subject was practised by the Alexandrian school in the times of the Ptolemies. Erasistratus and Herophilus, pupils of Chrysippus of Cnidus, experimented upon six hundred condemned criminals handed over to them by Ptolemy Soter. They opened the abdomens of these unfortunates to study the movements of the colon and those of the muscle of the diaphragm on the inspiration of air; they also opened the chests of some to study the cardiac movements. Their conduct, however, met with the reprobation of their contemporaries. Celsus and Galen reproached Herophilus with "cruel and useless sacrifices" of human feeling, while Tertullian called him roundly "an executioner who gave lingering death with refined cruelty." The court physicians of Attalus (King of Pergamus) and Mithridates (King of Pontus) were authorised in virtue of their office to try poisons upon criminals, and were accused by their jealous colleagues of pluming themselves upon their privileges, while less favoured practitioners were compelled to content themselves with cocks and dogs to experiment upon.

One of the earliest records of the use of animals for the purpose of physiological experiment is to be found in a document, still preserved in the Venetian secret archives, which bears the date 1432. It states: "Trial has been made on three porcine animals of certain venoms found in the chancery sent very long ago from Vicenza, which have been proved not to be good."

Brassavola of Ferrara studied little-known and doubtful remedies by testing their effects on criminals, and Fallopius, his pupil, who eventually made such important physiological discoveries, followed his master's example. It is recorded that Cosmo de Medici, Grand Duke of Tuscany, on one occasion ordered the magistrates of Pisa to hand over two men to Fallopius, "in order that he may put them to death in whatever way he pleases, and then anatomise them." Fallopius, however, seeing the men were condemned to death, seems to have acted with both dignity and humanity. He gave them each eight grains of opium; one died and the other recovered. Cosmo pardoned the latter unfortunate, but, if we may believe contemporary records, Fallopius did not: he gave the man eight grains more, and this time he died.

At Bologna, poisons were secretly administered to criminals to obviate the perturbing influence of fear upon natural toxic effects. Arsenic was employed in the same way at Mantua and Florence. Ambroise Paré, the eminent French surgeon, was invited by Charles IX. to try the alleged antitoxic action of the calculi found in the intestines of oxen, and known as bezoar, upon a man condemned to be hanged. What the poison was which was administered in this case is not stated, but the victim suffered so cruelly that he declared he would have preferred death upon the gallows a hundred times over. Even in the eighteenth century François Ranchin, Professor and Chancellor of the Faculty of Montpellier, wrote that experiments upon human beings were worthy of approval and had been held in high honour by the ancients.

English surgeons in days gone by were willing to avail themselves when the opportunity afforded to experiment on a condemned criminal. In 1731 a man named Charles Ray was reprieved on condition that William Cheselden, the famous anatomist and surgeon, should perforate the drum of his ear in order to ascertain if it would cause deafness. The unfortunate subject, however, was taken ill with fever before the experiment could be performed, and the operation was abandoned. Again, in 1763, another condemned man was offered a reprieve on condition that he consented to have one of his legs amputated to test the power of a new styptic. Fortunately, perhaps, for him, he died before the experiment could be performed. Four years later one John Benham is reported to have been reprieved for a similar purpose, but when Pierce, the inventor of the styptic, waited upon the Secretary of State to make arrangements, he was informed that his Majesty the King was of the opinion that it was quite improper to try such an experiment.

